# PPARs and HCV-Related Hepatocarcinoma: A Mitochondrial Point of View

**DOI:** 10.1155/2012/605302

**Published:** 2012-08-16

**Authors:** Francesca Agriesti, Tiziana Tataranni, Vitalba Ruggieri, Nazzareno Capitanio, Claudia Piccoli

**Affiliations:** ^1^Laboratory of Pre-Clinical and Translational Research, IRCCS, Centro di Riferimento Oncologico della Basilicata, 85028 Rionero in Vulture (Pz), Italy; ^2^Department of Clinical and Experimental Medicine, University of Foggia, 71100 Foggia, Italy

## Abstract

Hepatitis-C-virus-related infective diseases are worldwide spread pathologies affecting primarily liver. The infection is often asymptomatic, but when chronically persisting can lead to liver scarring and ultimately to cirrhosis, which is generally apparent after decades. In some cases, cirrhosis will progress to develop liver failure, liver cancer, or life-threatening esophageal and gastric varices. HCV-infected cells undergo profound metabolic dysregulation whose mechanisms are yet not well understood. An emerging feature in the pathogenesis of the HCV-related disease is the setting of a pro-oxidative condition caused by dysfunctions of mitochondria which proved to be targets of viral proteins. This causes deregulation of mitochondria-dependent catabolic pathway including fatty acid oxidation. Nuclear receptors and their ligands are fundamental regulators of the liver metabolic homeostasis, which are disrupted following HCV infection. In this contest, specific attention has been focused on the peroxisome proliferator activated receptors given their role in controlling liver lipid metabolism and the availability of specific pharmacological drugs of potential therapeutic utilization. However, the reported role of PPARs in HCV infection provides conflicting results likely due to different species-specific contests. In this paper we summarize the current knowledge on this issue and offer a reconciling model based on mitochondria-related features.

## 1. PPARs and Cancer

The peroxisome proliferator-activated receptors (PPARs) are transcription factors that translate nutritional signals into specific gene-expression patterns that control cellular differentiation, development, metabolism (carbohydrate, lipid, protein), and tumorigenesis. There are three members of the PPAR family: PPAR*α*, *γ*, and *δ* (aka PPAR*β*), which have tissue-specific distributions [1]. Each PPAR initially binds a ligand and then heterodimerizes with the retinoid X receptor (RXR) before the complex binds to DNA sequences referred to as peroxisome proliferator hormone-response elements (PPREs), which are generally found in the promoter region of PPAR-targeted genes [2]. This action of heterodimerization and binding to PPREs is further modulated by the presence of coactivator and corepressor proteins. The ligands for the PPARs consist in a range of metabolites, including certain free fatty acids, eicosanoids, and xenobiotics [1], referred as to peroxisome proliferators (PP) able to differentially modulate PPAR-regulatory activities.

PPAR*α* is the most abundant nuclear receptor in the liver especially in hepatocytes [3], and it has been identified as the master regulator of hepatic metabolism [4]. When activated, PPAR*α* upregulates *β*-oxidation and thus promotes lipid clearance. PPAR*α* has mostly been linked to fatty acid metabolism but it plays a role also in glucose metabolism. PPAR *γ* is expressed particularly in adipose tissue where it initiates the differentiation cascade in preadipocytes. Among its known target genes are adipocyte fatty acid-binding protein and fatty acid synthase, which are effectors of lipid accumulation during adipogenesis. Even if mainly expressed in adipocytes, also PPAR*γ* is involved in metabolism of hepatic cells. In contrast to PPAR*α* and *γ*, the function of PPAR*δ* is relatively unknown. PPAR*δ*, also known as PPAR*β*, NUC1, and FAAR, is expressed in a wide range of tissues, but progress in understanding the function of this protein has been hampered by the lack of selective ligands. PPAR*δ* has recently been implicated in a variety of physiological and pathophysiological processes such as embryonic implantation, wound healing, inflammation, cancer, and osteoporosis. Exposure of rodents to PPs leads to hepatomegaly, peroxisome proliferation, and an increase of fatty acid catabolism as a result of enhanced expression of genes involved in lipid transport and fatty acid *β*-oxidation [5, 6]. Accordingly, PPARs are involved in regulating other physiological processes such as cell proliferation, apoptosis, inflammation, oxidative stress, and differentiation. Although all these functions might contribute to the influence of PPARs in carcinogenesis, whether PPARs function as tumor suppressor or as oncogenes in cancer is still unclear.

Long-term administration of PPAR*α* ligands causes liver cancer in mice and rodents, an effect that is dependent on PPAR*α*, as PPAR*α*-null mice are refractory to the hepatocarcinogenic effect of PPAR*α* agonists [7]. Moreover, chronic exposure to synthetic PPARs agonists results in sustained activation of PPAR*α* and transcriptional activation of its responsive genes that affect intermediary liver metabolism leading to oxidative stress-induced DNA damage in liver [8, 9]. In response to ligand activation, the induction of the peroxisomal *β*-oxidation enzyme acyl CoA oxidase (ACO), as a result of peroxisome proliferation, increases intracellular levels of H_2_O_2_ leading to oxidative stress and/or generation of lipid peroxides or free radicals that could damage macromolecules [8]. In addition, it has been reported that mitochondrial dysfunction is also responsible for the oxidative stress induced by PPAR*α* ligands activity. Those ligands could disrupt mitochondrial electron respiratory chain at the level of the NADH cytochrome c reductase causing a compensatory shift in the metabolic state, which results in (i) preferential use of lipids through glycerol catabolism via mitochondrial FAD-dependent glycerol-3-phosphate dehydrogenase and (ii) stimulation of fatty acid *β*-oxidation via electron-transferring flavoprotein. The increase in free radical oxygen species resulting from stimulated peroxisomal *β*-oxidation may further increase the oxidative stress that results from complex I inhibition and thereby contributes significantly to the observed carcinogenic properties of PPAR ligand in rodents, particularly in liver [10]. Hypoxia in the tumour microenvironment is a common feature of solid tumours and known to stimulate mitochondrial release of reactive species of oxygen (ROS) able to function as important secondary messengers in signalling transduction. The increased ROS response can promote tumor growth and cell survival through activation of the hypoxia inducible factor 1*α* (HIF-1*α*) [11]. Interestingly, HIF-1*α* increases the expression of GLUT1 and other genes encoding glycolytic enzymes [12]. It has been demonstrated that hepatoma cell growth is dependent on the cellular redox state and that ROS could regulate glycolysis through HIF-1*α*. In fact, ROS levels directly regulate the hexokinase II (HKII) protein expression and lactate dehydrogenase (LDH) activity [13]. As redox level is able to modulate the tumour glycolysis in hepatoma cells, this mechanism could be exploited to selectively kill tumour cells through interference in energy pathways.

Liver cancer, as well as other solid tumours, shows an upregulation of the glycolytic activity in order to escape from the severe hypoxia characterizing the tumour microenvironment. Moreover, even under normoxic condition most transformed cells exhibit a robust dependence on glycolysis for energy production. This property, although long known as “Warburg effect,” still remains to be fully elucidated [14, 15]. The predominance of anaerobic/aerobic glycolysis leads to conversion of its end-product pyruvate to lactate, which is secreted into the blood, instead of completing oxidation [16]. Increased glucose uptake and metabolism, due to increased levels of glucose transporters (Gluts) and of HKII, correlate with poor prognosis of many tumor types [17], supporting the notion that metabolic alterations may contribute to the malignant phenotype [18].

Kroemer and Pouyssegur [17] showed a significant correlation between ^18^F-fluoro-2-deoxy-D-glucose (^18^F- FDG) uptake, evaluated through positron emission tomography (PET), and the expression of Glut2 and HKII. Another clinical study [19] demonstrated a high correlation between GLUT1 expression and Ki-67, a prognostic marker of proliferation. Moreover, GLUT1, absent in normal liver as well as in most of human HCC tissues [20], is upregulated in HCC with specific high proliferative activity and over expressed especially in hypoxic regions [19].

Recently, a DNA damage response-signaling network has been proposed as novel mechanism for PP-induced hepatocyte proliferation and hepatocellular carcinoma [21]. Accordingly several genes involved in cell cycle or DNA damage repair, such as *Chek1*, *Prkdc*, *Mcm*, and *Rad51*, were significantly induced in a PPAR*α*-dependent manner. It is postulated that PPAR*α*-induced-DNA damage repair is due to oxidative stress. Treatment of rodents with PPs induces expression of genes encoding enzymes involved in peroxisomal and mitochondrial fatty acid *β*-oxidation, which produces ROS as byproducts [8, 22, 23]. Previous studies also reported that several oxidative stress-related genes were upregulated upon PP challenge, such as Txnip, Sod2, Gpx2, and Cat [22, 23].

Collectively, these studies revealed the involvement of oxidative stress in the multiple effects of PP induced hepatocarcinomas. Interestingly, oxidative stress can activate a variety of transcription factors including PPAR genes [24] and this could be a novel link explaining their role in hepatic glucose homeostasis as well in liver carcinogenesis.

Activation of PPAR*α* also leads to increased proliferation and inhibition of apoptosis and when this occurs in a DNA-damaged cells, it is thought to lead to proliferation of initiated cells that ultimately progress to a liver tumor. This effect is supported by observation in PPAR*α*-null mice that are refractory to all these changes in response to long-term ligand-feeding studies [25, 26]. Whereas it is clear that PPAR*α*-agonists lead to increased cell proliferation and inhibition of apoptosis, the specific target genes mediating these events remain unidentified. Increased cell proliferation and inhibition of apoptosis are clearly causally linked to PPAR*α* agonist treatment and hepatocarcinogenesis. Since increased cell proliferation can influence both initiation and promotion events, the precise role of these changes is less clear. However, strong evidences causally link changes in cell proliferation and apoptosis to PPAR*α* agonist-induced hepatocarcinogenesis [27].

Thus, the metabolic changes along with the anti-apoptotic effects of PPARs activation contribute to oxidative DNA damage and increase hepatocellular proliferation leading to liver cancer development [2, 28].

Although the mode of action for the hepatocarcinogenic effect of PPAR*α* agonists has been determine in mice and rodents model, it is not clear if chronic administration of PPAR*α* ligands leads to tumorigenesis in humans. Different levels of regulation of PPAR*α*-induced response may account for this discrepancy among species. First of all, PPARs ligands show an intrinsic difference in their capability to induce maximal PPAR*α* activation and peroxisome proliferation whose induction, in part, can be dose dependent, but not a species-specific event. Furthermore, tissue levels of expression of the PPAR*α* receptor may explain the differences seen among animals and humans. PPAR*α* levels appear to be lower in human livers as compared to rodent livers and have been proposed to account for reduced response of human liver to peroxisome proliferation and tumors development. Being the PPAR*α* transactivational response also regulated by the nature of its recognition sequence presents in the promoter region of responsive genes, species differences in these promoters regions may account for some of the differences observed in response to treatment with PPAR*α* ligands. Finally, a differential expression of certain coactivator proteins necessary for PPAR*α*-mediated transactivation and different expression levels and activity of PPAR*α* target genes may contribute to the variable response among different species to treatment with PPAR*α* ligands. When the transcription coactivator complex PBP/Med is disrupted in mouse, liver, hepatocyte population fails to show cell proliferation and induction of peroxisomal *β*-oxidation enzymes exhibiting abrogation of peroxisome proliferation and other pleiotropic effects of treatment with PPAR*α* ligands. These data indicate that PBP is essential for PPAR*α* ligand-induced hepatocyte proliferation and tumorigenesis [29].

Recent data from studies using PPAR*α*-humanized mice (that express a human PPAR*α* gene in a PPAR null background) offer a new explanation for the species difference between rodents and humans in response to peroxisome proliferators (PPs) mediated by peroxisome proliferator-activated receptor PPAR*α*. It has been shown that activation of PPARs by its agonists, although causes overexpression of genes involved in lipid catabolism in both wild type and humanized mice, determines tumors development and hepatocellular proliferation only in wild-type mice [28, 29]. Moreover, mice expressing the human PPAR*α* protein do not exhibit a downregulation of the let7C miRNA, which in turn regulates the repression of c-Myc expression [30]. Thus the induced stability of Myc protein (in mice, but not in humans) might contribute to increased mitogenic signaling that causes hepatocyte proliferation in mouse model.

Despite the above-mentioned role of PPAR*α* in liver tumorigenesis, the role of PPAR*γ* in the onset and treatment of cancer has been focus of recent attention. Ligand activation of PPAR*γ* is associated with differentiation and inhibition of proliferation in the normal and malignant cells. PPAR*γ* agonists inhibit the proliferative activity of neoplastic cells, suppress the growth of human tumor xenografts in nude mice [31, 32] and reduce the frequency of spontaneous and carcinogen induced pre-neoplastic and neoplastic lesions in animals [31, 33], which is indicative of the tumor suppressor effects of PPAR*γ* [31]. The antitumorigenic effect of PPAR*γ* agonists in several liver cancer cell lines has been previously demonstrated [34, 35] although there have been no studies to mechanistically define the role of PPAR*γ* in hepatocarcinogenesis. In a recent work, by using a diethylnitrosamine (DEN)-induced murine model of HCC, it has been demonstrated that the loss of one PPAR*γ* allele significantly enhanced liver carcinogenesis. Accordingly, previous studies have been reported that human HCC displays impaired PPAR*γ* expression [34]. Moreover PPAR*γ* suppresses tumor cell growth through reducing cell proliferation and inducing G2/M phase arrest, apoptosis, and upregulating the putative suppressor gene, growth differentiation factor-15. Thus, PPAR*γ* has been proposed as a tumor suppressor gene in the liver [34].

In striking contrast another study unveils the regulation of TGF-*β* signaling (well known to inhibit hepatocyte proliferation and induce apoptosis) by cPLA2*α* and PPAR*γ* as an important mechanism for control hepatic cell growth and hepatocarcinogenesis [36]. In particular it has been described that PPAR*γ* signaling pathways counteracts TGF-*β* mediated inhibition of primary and transformed hepatocyte growth. The study has shown that TGF-*β* regulates the growth of primary and transformed hepatocytes through concurrent activation of Smad-mediated gene transcription and phosphorylation of cPLA2*α* suggesting that the level and activation status of cPLA2*α*/PPAR-*γ* signaling in hepatic cells likely represents a key factor that determines the cellular response to TGF-*β*. It is possible that activation of cPLA2*α*/PPAR-*γ* signaling may in part explain the loss of responsiveness of neoplastic cells to the anti proliferative actions of TGF-*β* (due to suppression of Smad2/3 activity) [36].

Agonists for PPAR isoforms induce many physiological changes and their oncogenicity seems to depend on the oxidative stress caused by peroxisome proliferators as well as on their ability to alter balance between cell proliferation and death. These are good reasons to suggest that PPARs agonists could be potential candidates for treating and preventing cancer. PPAR*α* remains a viable target for the treatment and prevention of cancer because of evidence indicating that humans are refractory to the hepatocarcinogenic effects of PPAR*α* agonists. PPAR*γ* also remains a potential target for the treatment and prevention of cancer, in particular for PPAR*γ* agonists with good safety profiles.

**Figure 1:**
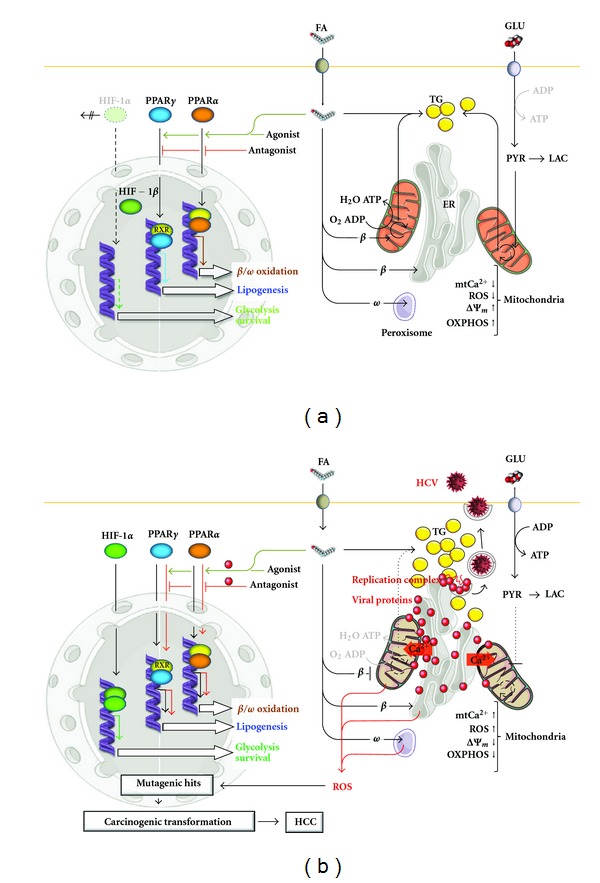
Schematic representation of the interplay between deregulation of the PPARs pathways and the HCV-mediated dysfunctions of mitochondria. (a) Normal noninfected condition. (b) HCV-infected condition Glu, glucose; Pyr, pyruvate; Lac, lactate; HIF-1*β*, hypoxia inducible factor 1*β*; RXR, retinoid X receptor.

However, the complexity of PPAR regulation and the effects resulting from receptor activation impose considerable research and drug discovery efforts to fully delineate the potential of targeting PPARs for the treatment and prevention of cancer.  Figure 1 illustrates schematically the main points discussed in this paragraph. The main mitochondrial alterations in in Figure 1(b) are highlighted in terms of intramitochondrial Ca^2+^ level (mtCa^2+^), reactive oxygen species level (ROS), membrane potential (ΔΨ_*m*_), and oxidative phosphorylation efficiency (OXPHOS). The tight physical interactions of mitochondria with the endoplasmic reticulum (ER) and the therein HCV proteins localization are shown. An attempt is made to reconcile the conflicting species-specific results reported in literature concerning the role of PPARs in the HCV-related development of steatosis and hepatocellularcarcinoma in infected subjects. The evidence in human is a reduction/increase of PPAR*α*/PPAR*γ* activities (black arrows starting from PPARs) whereas in mouse the opposite holds (red arrows starting from PPARs). This causes in infected humans enhanced lipogenesis with accumulation of triglycerides (TGs) in form of lipid droplets, which are essential for stabilization of the viral replication complex and consequent viral particle maturation. To note, under this condition a proinflammatory state is also developed because of the derepressive effect of PPAR*α* upregulation on NF*κβ* expression (not shown, but see text). In mouse the deregulation of the PPARs reciprocal activities would lead to enhanced uptake and oxidation of fatty acid (FA) as well as of lipoproteins (not shown). However, the set mitochondrial dysfunctions hamper the mitochondrial *β*-oxidation fostering ER- and peroxisome-mediated *β*- and *ω*-oxidation. This causes enhanced oxidative stress which adds to that of mitochondria. Such a constitutive pro-oxidative setting might provide mutagenic hits and genome instability leading to cell transformation and HCC development. Moreover, also in mouse the low efficient extramitochondrial FA oxidation leads to intracellular accumulation of lipids and progressive steatosis. Consistently, both PPARS control the expression of the FA transporters across the cell membranes. The possible direct involvement of the HCV proteins in controlling the PPARs pathways and the potential therapeutic utilization of PPARs agonist/antagonist to correct PPARs deregulation following HCV infection is also illustrated. For clarity co-transcription/corepressor factors recruited by PPARs have been omitted (but see text). The contribution of the hypoxia inducible factor (HIF-1*α*) to the metabolic/bioenergetic adaptation by (normoxic) up-regulation of the glycolytic pathway is also shown.

## 2. HCV and Liver Cancer

Hepatitis C virus (HCV) infects hundreds of millions of people persistently, and induces a spectrum of chronic liver disease worldwide [37]. Chronic infection with the hepatitis C virus (HCV) is a major risk factor for the development of hepatocellular carcinoma (HCC). The pathogenesis of HCC in HCV infection has extensively been analyzed. However, it remains controversial in the pathogenesis of HCC associated with HCV as to whether the virus plays a direct or an indirect role. HCV is a positive strand RNA virus with a 9.6 kb genome encoding a single approximately 3000 aminoacid polyprotein. This is translated on the endoplasmic reticulum (ER) and is co- and post-translationally cleaved by host and viral proteases into 10 individual membrane-associated proteins comprising structural (Core, E1, E2) and non structural (p7, NS2-NS5B) proteins [38]. It has been demonstrated that the core protein of HCV has an oncogenic potential, indicating that HCV is directly involved in hepatocarcinogenesis, albeit other factors such as continued cell death and regeneration associated with inflammation would also play a role.

It is known that the core protein is able to induce ROS increase in liver. Thus, oxidative stress production in the absence of inflammation by the core protein would partly contribute to the development of HCC. Accordingly it has been reported an augmented production of oxidative stress along with the activation of scavenging system, including catalase and glutathione, in the putative pre-neoplastic stage with steatosis in the liver. HCV infection can induce a state of oxidative stress that is more pronounced than that observed in many other inflammatory diseases. Many different oxidative stress markers have been reported in hepatitis C patients, including lipid peroxidation products [39] and oxidized protein and lipid derivatives in the liver [40]. Consistently a greater degree of oxidative stress markers correlates with a severer disease [39] and successful eradication of HCV decreases oxidative stress markers [41].

The generation of hepatic oxidative stress is assessed to originate from mitochondrial dysfunction in HCV-infected hepatocytes [42] and see Figure 1(b). Numerous studies have, particularly, shown that the expression of the HCV core protein can increase ROS production at the mitochondrial level. Although the synthesis and maturation of HCV proteins occur at level of ER [43], a number of studies unveiled partial localization of some HCV proteins, notably core and NS3/4a, to the outer mitochondrial membrane [44, 45]. A specific sequence in the C-terminal portion of the molecule serves as a targeting sequence to the mitochondrial outer membrane [44, 46]. At the mitochondria level, a chain of events is initiated by core binding, which consists of increased Ca^2+^ uptake, increased mitochondrial superoxide production, oxidation of the mitochondrial glutathione pool, inhibition of the electron transport complex I activity, and sensitization of mitochondria to Ca^2+^- and ROS-induced membrane permeability transition. These effects have been observed in isolated mitochondria [47], cells line inducibly expressing the entire HCV open reading frame (U-2 OS human osteosarcoma human derived cells) [43, 48] and liver mitochondria derived from HCV transgenic mice [49, 50]. In addition to these direct effects on mitochondria, core protein has been shown to cause a state of ER stress and an increase in the efficiency of ER to mitochondria Ca^2+^ transfer. The resulting oxidized redox state has a number of potential consequences for liver function: it interferes with the antiviral innate immune response and potentiates fibrosis and carcinogenesis. By using both HCV-induced U-2 OS derived cells and HCV-infected Huh-7 cells, our group has shown that the expression of all HCV proteins causes indirect Ca^2+^ mediated deregulation of the mitochondrial oxidative metabolism. Our studies unveiled a marked intra-mitochondrial Ca^2+^ increase as the causative event leading to profound mitochondrial oxidative metabolism alteration following coordinate HCV protein expression [48].

High level of calcium can detach cytochrome *c* from the cardiolipin inner membrane [51] and activate the mitochondrial nitric oxide synthase with production of nitric oxide (NO) [52, 53] that is known to be an inhibitor of complex IV [54, 55] as well as of complex I [56] although by a different mechanism. The combination of these possible effects would result in an overload of harmful reducing equivalents throughout the respiratory chain complexes and in an extraproduction of ROS with respect to their basal level [57, 58]. Once exhausted the buffering antioxidant capacity of glutathione and other redox buffers, a self-fuelling cycle can be activated with further enhancement of reactive oxygen/nitrogen species and alteration of the mitochondrial homeostasis [59, 60]. In summary, increased Ca^2+^, ROS (O^•−^, H_2_O_2_), and RNS (NO^•^, ONOO^−^) can trigger PTP opening and cytochrome c release across the outer membrane, culminating in the actuation of the apoptotic program, [56, 61] thus favoring diffusion of virus infection. Alternatively, ROS may act as redox modulators in pro-survival signaling (i.e., the NF-*κ*B/JNK/STAT3 pathway), resulting in carcinogenic priming of the host cell [56, 62]. Pharmacological treatment with compounds able to restore mitochondrial Ca^2+^ homeostasis might prevent or even reverse the effects of HCV [48].

Using a transgenic mouse model in which HCC develops late in life after the preneoplastic steatosis stage, it has been further demonstrated that HCV shrewdly exacerbates oxidative stress by modulating both production and scavenging of reactive oxygen species. Accordingly,the core protein of HCV was shown to induce overproduction of ROS in the liver. Under excessive generation of ROS, HCV affects the steady-state levels of a mitochondrial protein chaperone, that is, prohibitin, leading to an impaired function of the mitochondrial respiratory chain with the production of further ROS. On the other hand, HCV compromises some of the antioxidant systems, including heme oxygenase-1 and NADH dehydrogenase quinone 1. Thus, HCV infection not only induces ROS but also hampers the antioxidant system in the liver, thereby exacerbating oxidative stress that would facilitate hepatocarcinogenesis.

Another study confirmed the accumulation of an ROS-mediated oxidative DNA damage in the progression of chronic liver damage to HCC and suggested that this correlates with induction of telomerase activity and, as a novel finding, with overexpression of miR-92, a microRNA that plays a role in both the apoptotic process and the cellular proliferation pathways [63].

Recently a new strategy by which HCV promotes development of hepatocellular carcinoma has been proposed. It has been shown that the core protein overcomes premature senescence provoked by the ROS inducer, H_2_O_2_, in human liver cells. For this effect, core protein downregulates the level of p16 via promoter hypermethylation and subsequently induces phosphorylation of Rb in the presence of H_2_O_2._ The potential of core to inactivate Rb and suppress H_2_O_2_-mediated cellular senescence was abolished when levels of p16 were recovered by either exogenous complementation or inhibition of DNA methylation [64].

The core protein has also been found to interact with some cellular proteins, such as the retinoid X receptor (RXR)-*α*, which play pivotal roles in cell proliferation and metabolism [65]. The mitogen-activated protein kinase (MAPK) cascade is also activated in the liver of the core gene transgenic mouse model. The MAPK pathway, which consists of three routes, c-Jun N-terminal kinase (JNK), p38, and extracellular signal-regulated kinase (ERK), is involved in numerous cellular events including cell proliferation. In the liver of the core gene transgenic mouse model prior to HCC development, only the JNK route is activated. Downstream of the JNK activation, transcription factor activating factor (AP)-1 activation is markedly enhanced [65, 66]. Far downstream, both the mRNA and protein levels of cyclin D1 and CDK4 are increased. The suppression by HCV core protein of the inhibitor of cytokine signaling (*SOCS*)-*1*, a tumor suppressor gene, may also contribute to hepatocarcinogenesis. Thus, the HCV core protein modulates the intracellular signaling pathways and gives an advantage to hepatocytes for cell proliferation. Such an effect of the core protein on the MAPK pathway, combined with that on oxidative stress, may explain the extremely high incidence of HCC development in chronic hepatitis C.

Interestingly, it has been found that HCV core protein enhances Wnt/*β*-catenin signaling activity (in core-expressing hepatoma cells), whose overactivation is considered a major factor in oncogenesis. HCV core protein significantly enhances T-cell factor- (Tcf-) dependent transcriptional activity induced by Wnt3A in HCC cell lines. Additionally, core protein increases and stabilizes *β*-catenin levels in hepatoma cell line Huh7 through inactivation of GSK-3*β*, which contributes to the upregulation of downstream target genes, such as c-Myc, cyclin D1, WISP2, and CTGF. Also, core protein increases cell proliferation rate and promotes Wnt3A-induced tumor growth in the xenograft tumor model of human HCC [67]. The HCV 3a Core protein has also been found to downregulate the gene expression of caspases (3, 8–10) and p53, which are involved in apoptosis. Moreover, HCV-3a Core gene showed a stronger effect in regulating protein level of p-Akt as compared to HCV 1a Core accompanied by enhanced cell proliferation in Huh-7 cell line. Thus, it has been concluded that reduced expression of cellular genes involved in apoptosis, increased p-Akt (cell survival gene), and enhanced cell proliferation in response to HCV 3a core confirms antiapoptotic effect of HCV 3a Core gene in Huh-7 that may lead to HCC [68].

Novel insights into the pathogenesis of chronic hepatitis C and, possibly, the HCV-related development of hepatocellular carcinoma were provided by the observation that HCV induces normoxic stabilization and trans-activating upregulation of the hypoxia inducible factor (HIF) [69]. This result was obtained in different *in vitro* cell system and in human HCV-infected patient. The stabilization of HIF seemingly did not relate to the action of a specific viral protein but was rather due to accumulation of intermediates of the dysregulated mitochondrial metabolism. HIF plays a recognized role in adapting the cell to stressing conditions by upregulating the glycolytic pathway and providing pro-surviving features.

The combination of these pathways, collective with HCV-associated alterations in lipid and glucose metabolism (see below and Figure 1(b)), would lead to the frequent development of HCC in persistent HCV infection.

## 3. HCV and PPARs

 PPARs are master regulators of lipid and glucose homeostasis, inflammation, cell differentiation, and proliferation, processes intricately involved in HCV infection, and progression. HCV-mediated dysregulation of these processes influences the replicative efficiency of the virus [70–74]. PPARs are natural targets of HCV-related studies because of their abundant occurrence in the liver and involvement in processes known to be dysregulated by HCV. Because they regulate processes essential to the progression of chronic hepatitis-C, it is important to understand the interaction between HCV and PPARs pathways signaling.

 Studies on humans report impaired PPAR*α* activity in the livers of chronic hepatitis C patients [75]. Consequently, PPAR*α* mRNA and protein levels are significantly decreased in steatotic hepatitis C-infected livers as compared with non steatotic livers [76]. The HCV core protein contains RNA binding domains capable of suppressing the transcriptional activity of PPAR*α* [75] and, therefore has been indicated as a primary cause in the HCV-mediated downregulation of PPAR*α*. In addition to effects on metabolism, the decreased expression of PPAR**α**may be involved in the pathogenesis of HCV infection through an alteration of the protective effects of nuclear receptors against hepatic inflammation and fibrosis [77]. PPAR*α* downregulation observed in humans may also exacerbate HCV-induced inflammation [75, 78]. For example, the HCV core protein negatively regulates the inhibitory effect of PPAR*α* on nuclear factor kappa B (NF*κ*B) activity [78], thus activating NF*κ*B. From this point of view, PPAR**α** may represent new potential therapeutic targets in HCV infection.

 Conversely, studies in HCV core transgenic mice showed that the expression of core protein is associated with PPAR*α* activation. The core serves as a coactivator and nuclear stabilizer of PPAR*α* and may trans-activate PPAR*α* through ERK1/2 activation and p38 MAPK phosphorylation [79]. Although apparently counterintuitive, PPAR*α* upregulates genes involved in the generation of ROS through activation and induction of acyl CoA oxidase (AOX) and cytochrome P450 4A1. This causes increased microsomal and peroxisomal *β* and *ω* oxidation, respectively, which can cause oxidative damage of the mitochondrial membrane thereby impairing the mitochondrial *β*-oxidation and leading to fatty acid accumulation in hepatocytes [79, 80]. PPAR*α* also increases expression of fatty acid transporters, promoting fatty acid influx and leading to further PPAR*α* activation by acting as PPAR ligands [79, 80]; this helps to explain the role of PPAR*α* in HCV-induced steatosis in the animal model.

 PPAR*α* is also involved in the development of HCV-related HCC in animal models. PPAR*α* ^+/+^/HCV core transgenic mice develop HCC at a rate of about 30% higher than PPAR*α* ^+/−^/HCVcore or PPAR*α* ^−/−^/HCV core transgenic mice [79, 81]. This may be due to the involvement of PPAR*α* in ROS generation that subsequently leads to increases in oxidative DNA damage, predisposing hepatocytes to malignant transformation, and indicates that not the presence but the persistent activation of PPAR*α* would be important in hepatocarcinogenesis by HCV core protein. Moreover, PPAR*α* activation in mice also leads to increase in cell division by altering cyclins and cyclin-dependent kinases (CDK) expression without subsequent increase in apoptosis [79]. In general, PPAR-*α* acts to ameliorate steatosis but in the presence of mitochondrial dysfunction, which is also provoked by the core protein, the core-activated PPAR-*α* may exacerbate steatosis. Persistent activation of PPAR-*α* with strong ligands such as the core protein of HCV could be carcinogenic in humans, although the low-affinity fibrate ligands are not likely associated with human cancers.

 Conflicting evidences have also emerged concerning the role of PPAR *α* in HCV replication upon study of its ligands. It has been shown that both PPAR*α* agonists and antagonists inhibit HCV replication [82, 83]. Bezafibrate, a PPAR*α* activator, is widely used to treat hyperlipidemia by reducing serum LDL, VLDL, and triglycerides. Fibrates may decrease HCV RNA titers in patients who were previously unresponsive to IFN therapy [72, 84]. This effect is attributable to its reduction of HCV RNA bound to LDL. It is also possible that PPAR*α*-mediated suppression of NF*κ*B is involved in HCV repression [72]. The repressive effects of an agonist are logical due to the anti-inflammatory as well as antilipogenic properties of PPAR*α*. However, PPAR*α* antagonists also make sense given the environment needed for viral replication. HCV replication takes place in membranous ER-derived complexes that associate with lipid droplets. HCV core induces changes in lipid metabolism [85, 86] as well as the formation and redistribution of these droplets [87, 88]. PPAR*α* antagonist 2-chloro-5-nitro-N-(pyridyl) benzamide causes hyperlipidemia and consequent disruption in the membranous structures and in the composition of lipid droplets (notably an increase in triglyceride content) in Huh7 cells. This causes changes in the localization of HCV RNA and disruption of the replication complex [79, 82]. However, similar effects (i.e., inhibition of HCV replication) have also been described following treatment with PPARa agonists [89]. Evidences are accumulating suggesting caution in interpreting effects of drugs which appear to have pleiotropic effects and that cannot be ascribed solely to PPARs as specific targets [10]. Moreover, it should be taken into account that contradictory results might stem from differences in the cellular environment altering directly or indirectly the effects on PPARs as through phosphorylation of the receptors or availability of cofactors.

 As with PPAR*α*, there is some controversy also about the interaction between PPAR*γ* and HCV. Several in vitro studies using the human HCC cell line Huh7 have linked HCV (specifically viral protein NS5A) with increased transcriptional activity of PPAR*γ* [90, 91] as well as increased recruitment of PPAR*γ* coactivator-1*α* (PGC1*α*) to the peroxisome proliferator response element [91]. Increased expression of PPAR*γ* mRNA has also been observed in human livers infected with HCV, with the highest levels in patients with HCV-associated steatosis [92]. PPAR*γ*-mediated up regulation of lipogenic genes is a relatively simple mechanism for HCV-related steatosis. However, HCV genotype 3a-mediated down regulation of PPAR*γ* in Huh7 cells has also been observed, leading to induction of suppressor of cytokine signaling 7 (SOCS-7), which is normally repressed by PPAR*γ*, and helping the virus to inhibit cytokine signaling and to escape the immune system [74]. SOCS-7 also plays a role in the development of insulin resistance, a well-known result of chronic HCV infection, by degrading insulin receptor substrate 1 in Huh7 cells [74, 93].

There is sufficient evidence that HCV infection affects PPARs-mediated pathways thus affecting the hepatic metabolism. The relationship between hepatitis C and nuclear receptors is undoubtedly complex and it is difficult to assemble the often-discordant findings into a comprehensive picture (Figure 1). However, what emerges clearly is the profound impact of nuclear receptor-regulated pathways on the critical steps of the viral life-cycle. In-depth understanding of the interaction may prove a crucial step in the development of treatment and prevention strategies.
